# Dye Clicked Thermoplastic Polyurethane as a Generic Platform toward Chromic-Polymer Applications

**DOI:** 10.1038/s41598-019-54832-5

**Published:** 2019-12-09

**Authors:** Eunbyeol Seo, Jihyun Choi, Bumjae Lee, Young-A Son, Kyung Jin Lee

**Affiliations:** 1 Department of Chemical Engineering and Applied Chemistry, College of Engineering, Chungnam National University, 99 Daehak-ro (st), Yuseong-gu, Daejeon 305-764 South Korea; 2 Department of Advanced Materials Engineering, College of Engineering, Chungnam National University, 99 Daehak-ro (st), Yuseong-gu, Daejeon 305-764 South Korea

**Keywords:** Chemistry, Engineering, Materials science

## Abstract

Chromic dyes responding against external stimuli are useful in various field of applications especially to colorimetric sensors. However, there have been several limitations in generic application because of its cost, stability and reliability. Here, we introduced highly functionalizable polymeric materials as a supporter covalently modified with controlled amount of chromic dyes. The photochromic organic dye (spiropyran) and highly functional thermoplastic polyurethanes (TPU) have been adopted as a representative example. Conventional polymeric solution processes such as film processing, wet-spinning, electrospinning and ink-writing are readily applicable because dye-TPU maintains its own solubility in various organic solvents. Additionally, since the concentration of dye on TPU are precisely controllable, these dye-TPU solution can be adopted in broad range of specific applications, such as secret coding, smart fabric, and chromic polymeric film layer.

## Introduction

Chromic dyes that change the original optical colors against external stimuli have received great attention in recent decades and there has been a great deal of effort to develop useful platform for colorimetric sensors signaling certain stimuli, i.e. ultraviolet light^1^, temperature^2^, pH^3^, and hazardous chemicals from environments^4^. Different types of chromic dyes have been reported so far, and are generally classified according to the type of stimulation, for example, thermos-chromic^5^, photo-chromic^6^, mechano-chromic^7^, chemo-chromic^8^ and so on^9^. Series of chemical structures have also been proposed to implement chromism such as spiropyran^10^, azo^11^, coumarin^12^ and rhodamine^13^ based structures. Tremendous efforts on these fields lead scientific and technological advances on handling and usage of chromic dyes for final applications. However, considering the unlimited possibilities on chromic dyes, there are still struggling issues for practical applications, mostly due to their cost, stability, reliability, and easy accessibility.

Of issues for final applications, development of proper supporters carrying chromic dyes will be the most important because the chromic dyes which are simply organic molecules are difficult to be utilized in practical applications by just themselves^14^. Therefore various pathways to incorporate chromic dyes have been suggested so far, but each of method requires tedious optimization process for final applications according to their type of dyes or carriers and application fields, which will be the greatest huddle for practical usage^15^. For example, conventional dying technology can be adopted to introduce these chromic dyes into fabric or clothes, but only relatively limited number of dyes can be applicable^16,17^. Especially, for colorimetric sensor applications, control of their concentration is the most important, which is difficult to achieve using each independent method^18^.

Among the candidates for supporter (or carrier) of chromic dyes (or organic dyes), polymeric materials provide great potential due to the easy processibility, accessibility and abundant functionality^19,20^. Essentially, most of typical applications for chromic dyes are depended on polymeric carriers^21,22^. Chromic dyes have been introduced into polymeric supporters by usually surface treatment or simple blending that obviously contains several issues in terms of their stability, controlling of concentration, and durability^23,24^. Therefore, it will be great if one can provide polymeric materials carrying tailored amount of chromic dyes via covalent bond with maintaining their processibility.

Although easy processibility and large amount of functional groups in chain will be the best advantages of polymers, simultaneous achievement of both properties are really challenging^25^. For example, as increasing amount of functional moiety (with desired molecules i.e organic dyes), the solvent property of polymers will be changed, and thus re-optimization of processing should be followed. Therefore, development of further functionalizable polymers with controllable solubility even after incorporating specific functional moieties will be really helpful for considering diverse applications. Herein, we report on synthesis of highly functional thermoplastic polyurethanes (TPU) having bunch of azide functional groups in side chain which can be covalently anchored by organic dyes with desired/tailored concentration and thus shows minimized changes in solvent properties after modification. Photochromic dyes (Spiropyran type) have been selected as a representative example. This organic dye can be covalently anchored on pendant azide of TPU via click chemistry with controlled concentration. Based on soluble TPU with functional dyes, we can prepare TPU based functional dye solutions with controllable concentration for final applications. Finally, we demonstrate several solvent processes of dye-TPU using conventional solution process such as film making, wet spinning, electrospinning, and ink-writing. Because chromic dyes are clicked with polymeric chains, no additional carriers or treatments are required for final usage. Several useful applications using these pre-controlled dye solutions have been demonstrated as well. These versatile platforms can be expanded to prepare polymer-based chromic applications such as smart windows, smart fabric, optical sensor patch for early notice against environmental changes, and security inks.

## Methods

### Materials

To synthesize azido TPU, epichlorohydrin (ECH) (≥99%), boron trifluoride THF complex (>99.5%), dibutyltin dilaurate (95%), and MDI (98%) were obtained from Sigma-Aldrich chemical Co. Methylene chloride (MC) (99.5%), THF (99.5%), N, Nʹ-dimethylformamide (DMF) (99%), and sodium azide (99%) were purchased from SAMCHUN Chemical Co. To fabricate spiropyran (SP) including alkyne group, 1-(2-Hydroxyethyl)-3,3-dimethylindolino-6′-nitrobenzopyrylospiran (>93.0%) was purchased from TCI (Tokyo Chemical Industry Co.). propionic acid (95%), DCC (99%) and 4-(Dimethylamino)pyridine (DMAP) (≥99%) were obtained from Sigma-Aldrich Chemical Co. For the click reaction between azido-TPU and spiropyran, copper (II) sulfate pentahydrate (99.0%) and sodium ascorbate (98%), which act as catalysts, were obtained from Sigma-Aldrich chemical Co.

### Synthesis of azido-TPU

Synthesis of azido-TPU was performed as reported previously.[26] Azido-TPU was synthesized with 3 steps. Firstly, poly(ECH-co-THF) diols was obtained by cation ring opening polymerization between ECH and THF. And chlorin group of poly(ECH-co-THF) diols were substituted to azide group. So, poly(GAP-co-THF) diols was obtained from azidation method. Finally, azido-TPU were fabricated from poly(GAP-co-THF) diols, MDI and BDO.

### Functionalization of spiropyran and click reaction with azido-TPU

Spiropyran including alkyne group are prepared to perform click reaction with azido-TPU. To functionalization spiropyran, the Steglich esterification method was adopted. Steglich esterification is one of the reactions between hydroxy group and carboxyl group and form ester bond in structure. MC (45 ml), spiropyran (0.15 g) and propiolic acid(0.04 ml) were mixed in flask. Then, DCC (0.13 g) and DMAP (1.00 mg) were added as the catalysts under stirring at 24 °C for 20 hrs. After reaction, the mixture extracted several times with 5 wt% sodium carbonate solution. The solution was evaporated to remove MC and dried for 24 hr. Then, spiropyran with alkyne group can be bonded to azido TPU via click reaction with controlled concentration. was performed click reaction with azido-TPU. Four different concentrations, roughly 90, 50, 30 and 10 mol% of SP-TPU were synthesized. Typically, in order to prepare the about 50 mol% SP-TPU, DMF (47.37 g), alkyne-spiropyran (0.25 g) and azido-TPU (5 g) were mixed in flask. Then, copper (II) sulfate pentahydrate (0.25 g) and sodium ascorbate (0.07 g) were added as the catalysts under stirring at 45 °C for 24 hrs. At the same amounts of azido-TPU and catalysts, the amount of alkyne-spiropyran varies from 90 mol% SP-TPU to 0.29 g, 30 mol% SP-TPU to 0.15 g, and 10 mol% SP-TPU to 0.07 g.

### Diverse processing

Based on TPU with functional dye, SP-TPU solution was prepared with controllable concentration for diverse applications such as electrospinning, film, ink-writing and yarn. SP-TPU was dissolved in THF/DMF (8:2 v/v) at 60 °C for electrospinning. The prepared 20 wt% of SP-TPU solution was loaded into syringes that were connected with 25 gauge needles (EFD, Korea) and pumped using a syringe pump (KDS100, KD Scientific Inc., US). The feed rate of SP-TPU polymeric solution was 0.2 ml/h. A voltage was applied by the power supply (SHV50R, Conver tech, South Korea) on the polymer solution and electrospinning condition was 0.8–12 kV. The ground electrode was linked to collector which was rotating aluminum foil. To obtain the SP-TPU films, spin-coating process was used with 10 wt% of 90 mol% of SP-TPU polymer solution in THF. 1 ml of polymer solution was dropped on the 27 × 76 mm of slide glass, and the spin-coating has been performed at 800 rpm for 10 seconds to obtain thin film. After dried at room temperature, the SP-TPU04 polymer film on slide glass was peeled. SP-TPU polymer solution was also used as ink for writing. To obtain polymer writable -ink, 5 wt% of SP-TPU polymer solution in THF was prepared. Finally, yarn form can be obtained by wet-jetting method. SP-TPU was dissolved in DMF at 70 °C for wet-jetting. The prepared 15 wt% of SP-TPU solution was filled into syringe that was interjoined with 25 gauge needles and pumped using a syringe pump with 1.8 ml/h rate. Then, pull the polymer solution from the needle to get the yarn under water. The obtained yarn will used as sewing to the fabrics. In order to observe UV chromism for each polymeric product, the fabricated non-woven fiber, film, yarn and ink-solution were UV-irradiated (365 nm UV LED) during 30 ~ 90 seconds.

### Material characterization

FTIR (Spectrum Two, PerkinElmer Inc., USA) and NMR Spectroscopy (^1^H-NMR; Avance™ III, Bruker Inc., USA) were used for confirming the spiropyran functionalized with alkyne. The morphology of electrospun SP - TPU fibers were observed using Scanning Electron Microscope (SEM; S-4800, Hitachi Co., Japan). In addition, the PL spectra (RF-6000, Shimadzu Co., Japan) were obtained to confirm tendency of concentrations of SP-TPU. ^1^H NMR (600 MHz, DMSO-*d*_6_, δ): 2.8 (s, 1 H, CH), 3.5 (t, *J* = 4 Hz, 2 H; CH_2_); TGA (TGA N-1000, Sinco, Korea) was used for thermal property analysis with bare TPU and SP-TPUs. The difference of SP-TPU film upon UV irradiation was observed by UV-Vis spectra (6600 UV-VIS, PhotoLab, Europe).

## Results and Discussion

### Synthesis of solution processible TPU with organic dye

**Figure 1:**
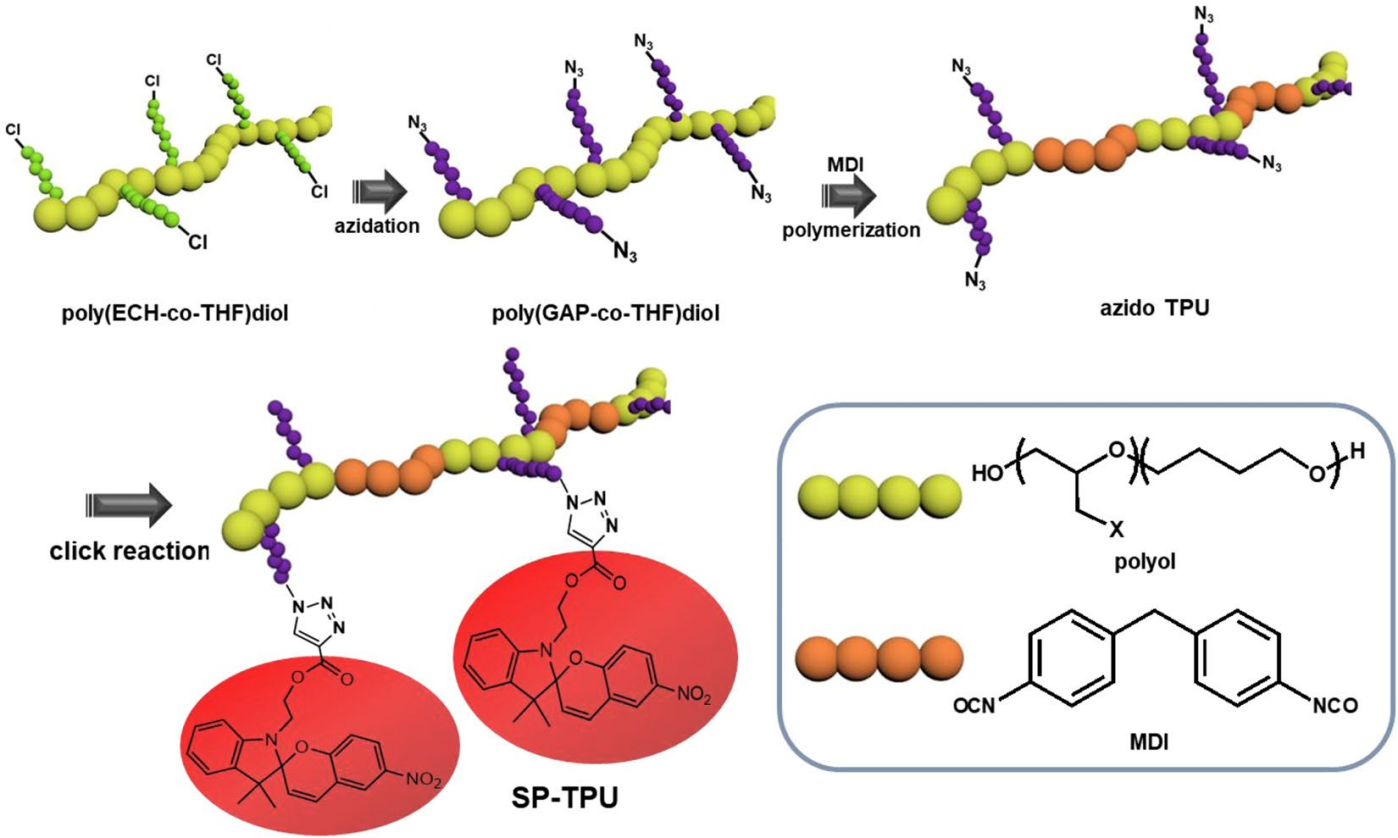
Schematic diagram of synthesis of dye-TPU.

Overall synthetic procedure to prepare highly functionalizable and solution processible spiropyran-TPU (SP-TPU) are schematically demonstrated in Fig. 1. Detailed synthetic methods have been presented in our previous reports^26^. In general, azide-diol has been firstly synthesized by S_N2_ substitution reaction of chloride and azide from poly(epichlorohydrin-co-tetrahydrofuran) (poly(ECH-co-THF)) obtained by cationic ring opening polymerization^27^. Adopting the several chain extenders such as 1,1ʹ-methanediylbis(4-isocyanatobenzene) (MDI) and butane-1,4-diol (BDO), azido TPU can be synthesized with controllable molecular weight range from 30,000 to 100,000. Here, as a representative example, we selected azido-TPU with average molecular weight of 60,000 (Fig. S1) that shows enough polymeric properties in various process. All the spectroscopic analysis of TPU have been displayed in our previous reports and supporting information as well (Fig. S1)^26^. After then, SP molecules are covalently anchored on pendant chain of polymer with desired concentration.

**Figure 2:**
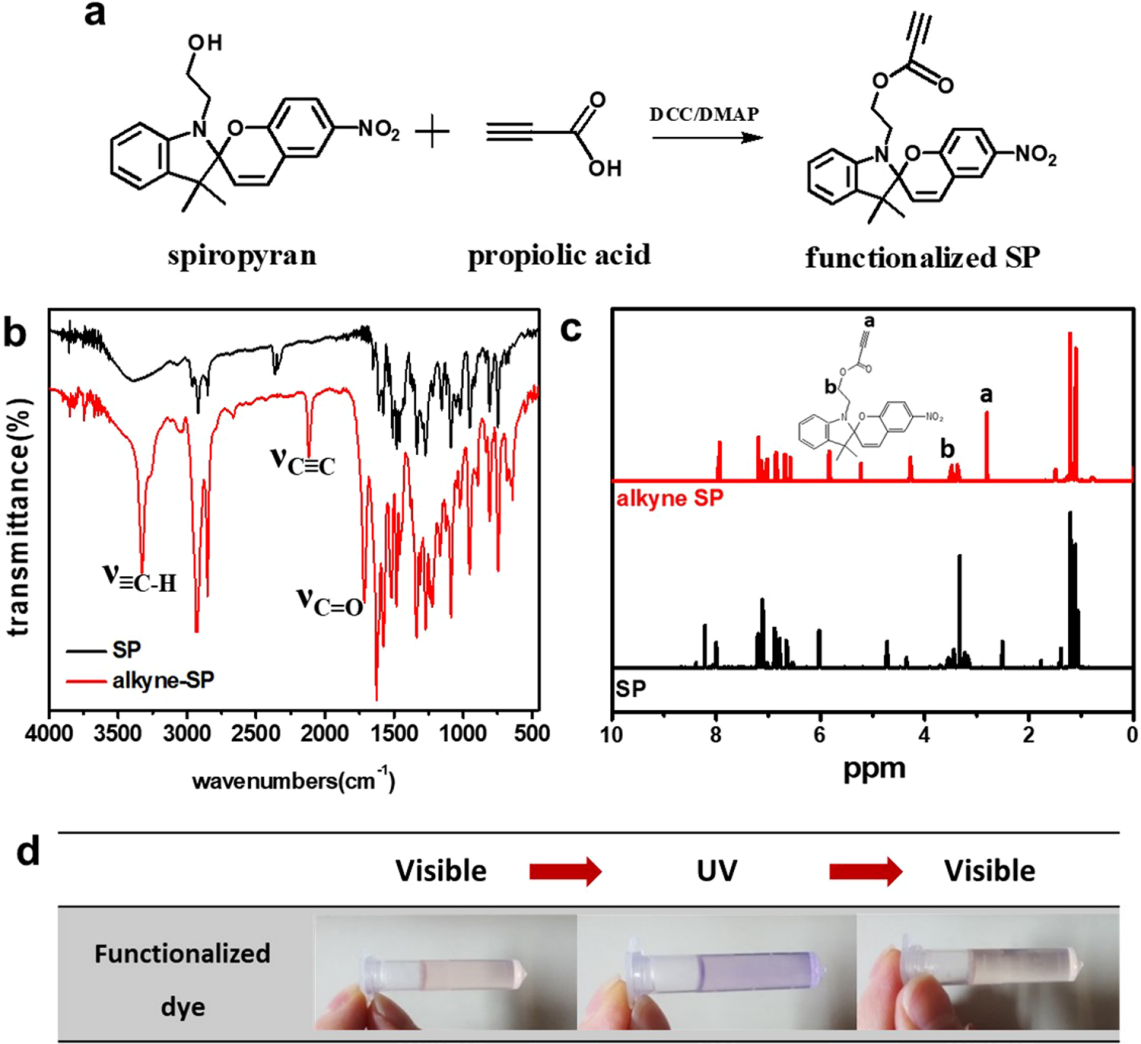
(**a**) Synthetic scheme of functionalized spiropyran, (**b**) FTIR spectra, (**c**) ^1^H-NMR spectra and (**d**) reversible color change image of alkyne SP.

In order to be applicable in azido-alkyne click chemistry, the SP molecules should be modified with triple bond as schematically described in Fig. 2a. Simple esterification between hydroxyl group of SP and carboxyl group of propiolic acid can make it possible to incorporate triple bond on chromic molecules without deterioration of chromic properties. The Fourier transform infrared (FTIR) and ^1^H Nuclear Magnetic Resonance (NMR) spectra (Fig. 2b,c) of SP and alkyne SP confirmed the successful functionalization of spiropyran with alkyne group. Compared to FTIR spectrum of original spiropyran, the C≡C bond streching peak at 2116 cm^−1^ and the ≡C-H bond at 3312 cm^−1^ were clearly observed in that of alkyne SP. In addition, the presence of C=O streching at 1720 cm^−1^ indicates the ester groups in structures. Furthermore, the ^1^H-NMR results of alkyne SP confirmed the sucessful esterification of SP with propiolic acid. The -C≡H peak of alkyne SP at 2.8 ppm appears unlike spectrum of original SP^28^. The methylene proton next to ester oxygen was observed at 4.2 ppm as well^29^. FTIR, ^1^H-NMR and additional GC/Mass spectra (Fig. S2) confirmed that the chromic moiety was not affected by esterification. Additionally, it is shown in Fig. 2d that the chromic properties of alkyne SP against UV are maintained. In tetrahydrofuran (THF) solution, the reversible color change of alkyne SP are observed.

**Figure 3:**
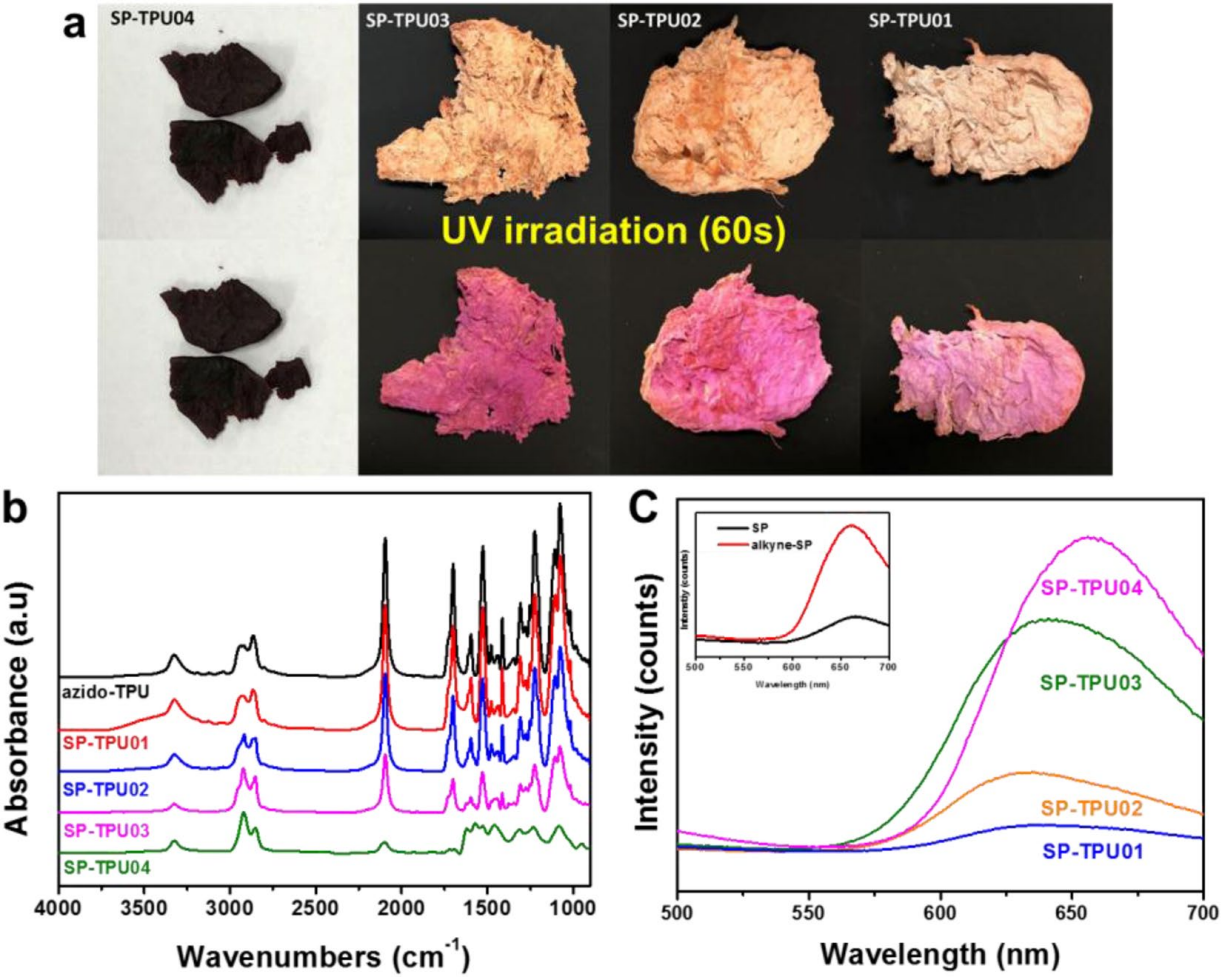
Quantitative control of dye: (**a**) photograph of three concentrations SP-TPU polymers with after UV irradiation, (**b**) ATR-FTIR spectra of azido-TPU and SP-TPU polymers and (**c**) PL spectra of SP-TPU polymers (inset: PL spectra of orginal SP and alkye-SP).

During the click reaction, tailored control of dye concentration on azido-TPU is possible. From an area of ^1^H-NMR spectra of azido-TPU, the molar ratio of MDI, BDO, glycidyl azide polymer (GAP), and THF constituting the TPU chain can be determined. Theoretically, one mole of TPU with 60,000 number molecular weight contains 210 mole of azido group. Here, we just prepared SP-TPU containing four different chromic dyes by controlling average substitution ratio of SP on azide group of azido-TPU with 60,000 molecular weight; roughly *c.a*. 90 (SP-TPU04), 50 (SP-TPU03), 30 (SP-TPU02) and 10 (SP-TPU01) mol% subtitution of azide groups on azido-TPU (Fig. 3a). The substitution ratio of alkyne SP against azido groups are roughly obtained by analysis of FTIR spectra (Fig. 3b). The degree of substitution was quantified by FTIR spectra with internal standard of C-H stretching band at 2800 ~ 3000 cm^−1^, which will be unchanged via additional click reaction. All the FTIR spectra have been normalized with fixed intensity of C-H stretching band, and then changing of peak area for azide band (2100~2200 cm^−1^) have been traced. Azide peak area for non-functionalized TPU will indicate zero-degree of functionalization meaning average 210 mole of azide groups will remain on the chain. In the case of SP-TPU04, peak area of azide band was 10% against non-functionalized TPU. Theoretically all of azido groups in single chain of TPU can be substituted with SP, which will be the best in final applications in terms of their color change. Here, fortunately all of SP-TPU above can be soluble in organic solvents (however, the maximum substitution ratio maintaining similar solubility to that of bare TPU should be varied according to type of dyes, molecular weight of TPU, and molecular structure of TPU (i.e, ratio of chain extender, molecular weight of initial diol, etc)). Here we just select above 4 different concentrations as standard solutions for demonstration and obviously all of these maintain their original solubility.

After UV-irradiation, the color of each polymer are readily distingusiable as denoted in Fig. 3a. Noted that the SP-TPU04 which contains the highest amount of alkyne SP does not present color difference after UV irradiation in bulk polymer form because of their strong original color. Different photoluminescence (PL) intensity can be obtained from each SP-TPU/THF solution with same polymeric concentration (10 w/v%) of SP-TPU (Fig. 3c). The PL spectra of SP and alkyne SP are identical as shown in inset of Fig. 3c and these PL properties are also maintained after TPU anchoring. It is noteworthy that peak positions are red-shifted as increasing the SP concentration in single TPU chain, which can be observed in PL spectra of single-dye solution with different concentration (Fig. S3). Additionally, thermo gravimetric analysis (TGA) have been carried out for the four types of SP-TPU polymer (Fig. S4a). The thermal stability is slightly decreased as increasing dye concentrations because of inherent weak thermal stability of dyes, implying again successful anchoring of SP dyes on TPU with tailored concentration. The mechanical properties of each polymer (casted into dog-bone shape) have been slightly changed as introducing SP dyes into TPU (Fig. S4b), but all of SP-TPU still contains elastic properties of TPU. These chromic dye-TPUs are quite generic platform because literally unlimited type of SP-TPU with desired degree of chromic can be synthesized by controlling the density of azido groups or molecular weight of TPU. Furthermore, these serise of synthetic method is not limited to SP dyes, but also can be extended to diverse type of chromic or organic dyes (Figs. S5 and S6).

### Broad range of application based on solution processible dye-TPU

**Figure 4:**
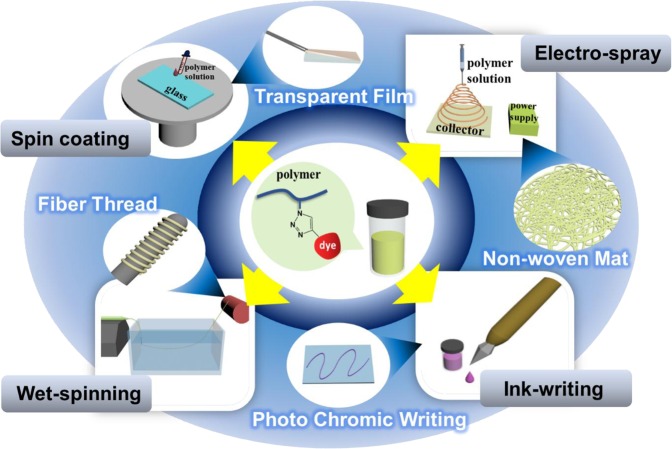
Schematic diagram of diverse process using SP-TPU polymer.

Results above indicate that we can prepare chromic dye anchored polymers with pre-defined chromic with desired processibility. Because their solvent property is basically same with bare-TPU, diverse types of SP-TPU products can be fabricated using different solution processing methods as schematically described in Fig. 4. Transparent films, fiber bundles, non-woven nanofibers and polymeric inks are prepared using spin-casting, wet-spinning, electrospinning and ink-writing (or theoretically ink-jetting is also possible) process, respectively. Noted that, for example, because we can synthesize SP-TPU with different chromic, we can also prepare polymeric inks with different pre-controlled colors. Using these diverse types of polymeric products having chromic properties, several useful applications can be considered without tedious optimization process for proper carrier.

**Figure 5:**
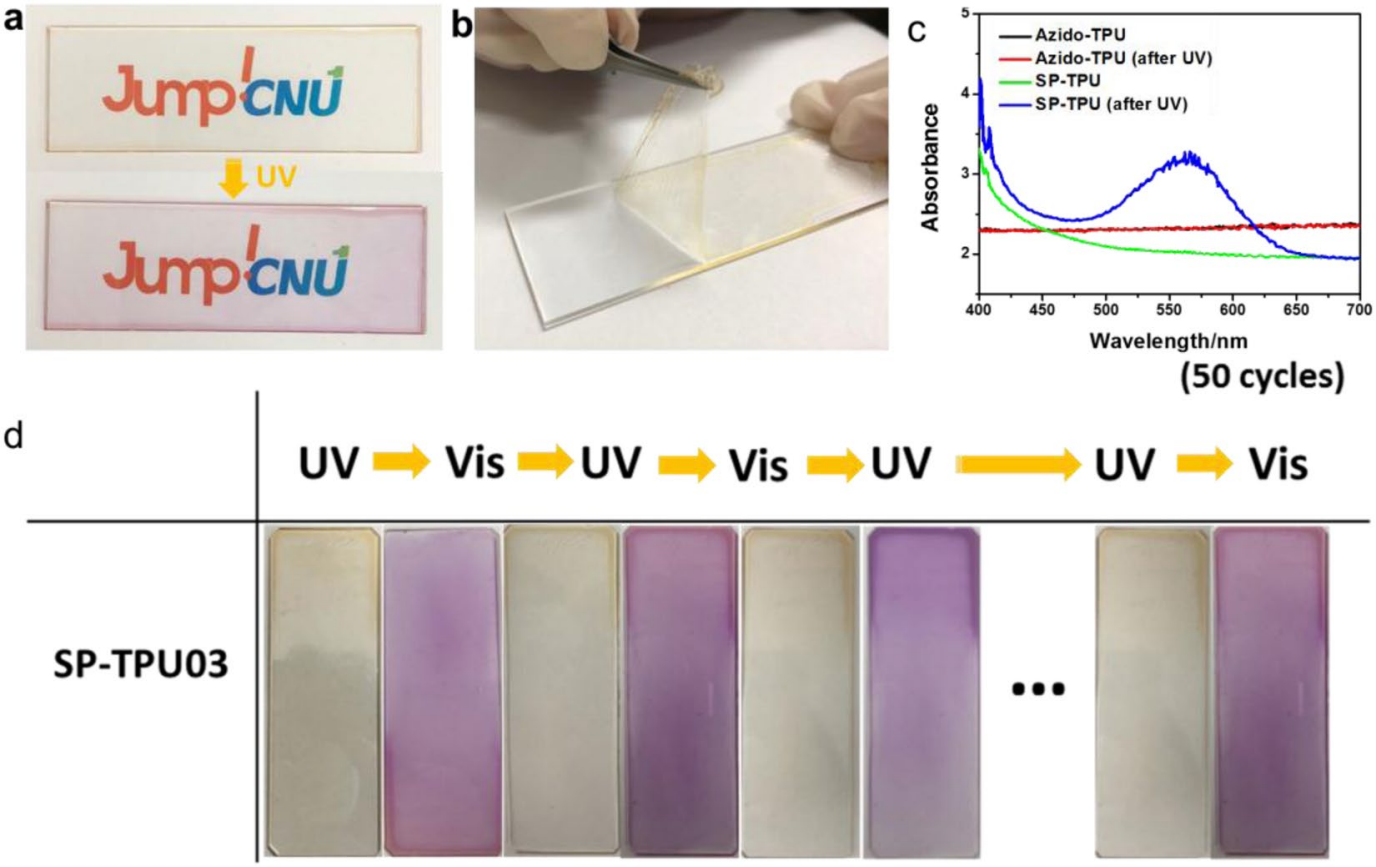
Film process: (**a**) photograph of spin-casted SP-TPU04 transparent films before and after UV irradiation, (**b**) the SP-TPU film peeled off from slide glass, (**c**) UV-Vis spectra before and after UV irradiation of Azido-TPU and SP-TPU03, (**d**) cycle test of color chages for SP-TPU03 films.

First of all, as presented in Fig. 5a, transparent polymeric films of SP-TPU (here, SP-TPU04) can be fabricated via simple spin-casting method. This film is not only transparent identical to bare TPU, but also is detachable from substrate as a free-standing films (Fig. 5b). Originated from photochromic dyes in polymeric chain, the films can be transformed into different color via UV-irradiation, which can be also confirmed by UV-Vis spectra of SP-TPU films before and after UV-irradiation (Fig. 5c). The reversibility is quite reasonable as indicated Fig. 5d, and over 50 cycles there is no remarkable reduction in performances, which can make it possible to consider diverse application fields such as smart window based on chromic platform^30^. In addition, these SP-TPUs maintain their original characteristics of bare-TPU including elastic properties as well, as shown in Fig. 5b.

**Figure 6:**
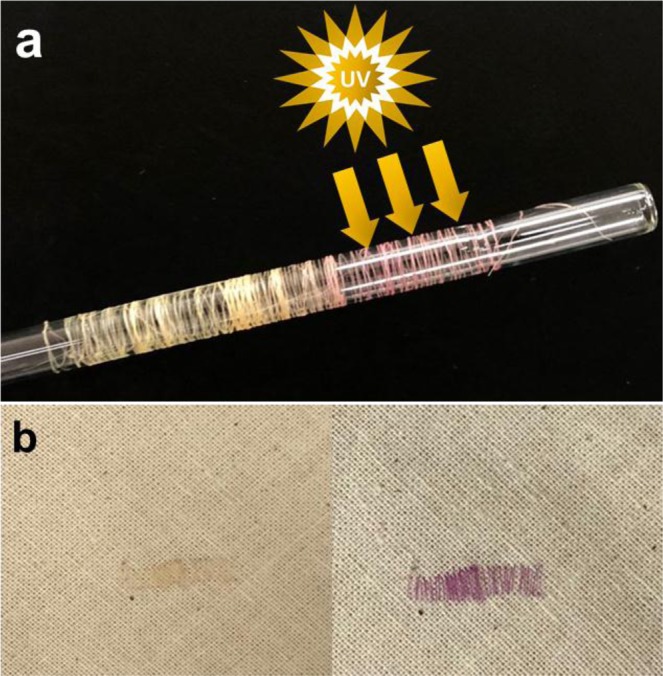
Wet-jetting process: (**a**) photograph of wound fiber on a glass rod and (**b**) stitched SP-TPU02 fiber on fabric before and after UV irradiation.

Noted that one of great advantages of this method will be their processibility with tailored chromic, so that one can prepare fabric thread and bundles bearing controlled chromic properties via wet-spinning methods (Fig. 6a)^31^. It is obvious that the photochromic properties of SP-TPU is maintained under processing condition, as described in Fig. 6a. These fabric threads can be adopted in conventional stitching or weaving process based on their sufficient mechanical properties (Fig. S4), and diverse fabric patterns can be introduced into fabric (Fig. 6b). This SP-TPU based fibers or fabric contains superior advantages to previous chromic fibers which is depended on conventional dying technology. In the SP-TPU, because the chromic dyes are covalently anchored onto polymer directly, they show great stability in terms of especially elution. In addition, the colors (or chromic) are pre-defined in synthetic procedure, and thus diverse types of chromic fibers in their color or type of chromism can be manufactured and supplied directly into weaving industry. Combined with i.e. 3D weaving machine^32^, one can produce smart fabrics with specific targets (such as chemo-sensor, thermos-sensor) or novel type of “fashion and design” fabrics using these chromic polymer platform^33^.

**Figure 7:**
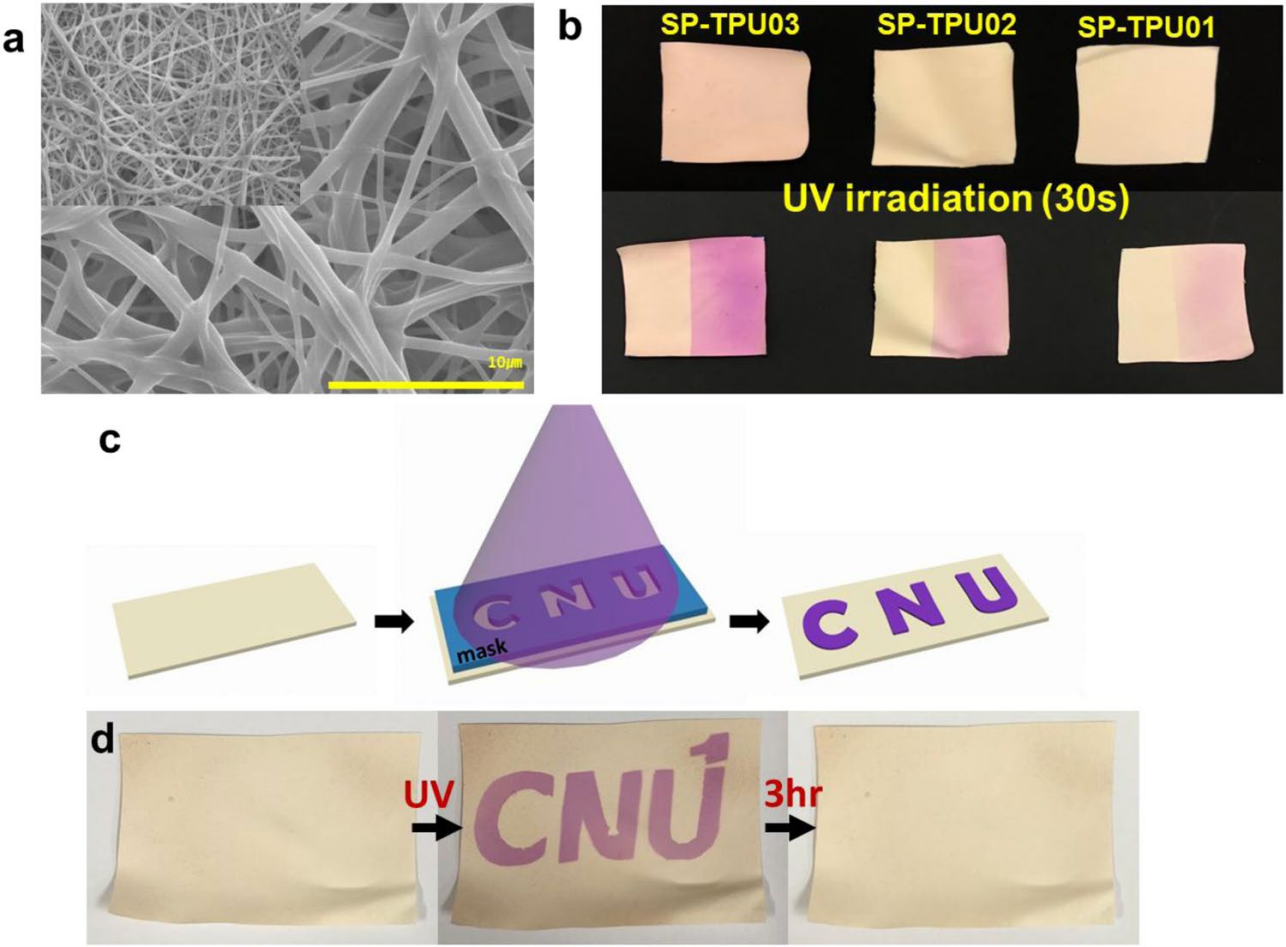
Electrospinning process: (**a**) SEM images of fiber, (**b**) photograph of three types of concentrations fiber mat after UV irradiation, (**c**) scheme of masking process on fiber mat and (**d**) reversible chromic patterning on SP-TPU02.

These chromic polymer platforms can be also adopted as colorimetric sensors against specific stimulus (i.e with UV-sensor using photochromic, chemo-sensor using chemochoromic, or even stretching sensor using mechanochromic) for an early warning system^34,35^. Important factors in these applications will be chrominance before and after exposure (must followed by this work afterwards for better application), and especially the surface areas. Smart patches with large surface area will be beneficial to fast detection of external stimulus^36^. Electrospinning is the one of best candidate to prepare non-woven nanofiber patch^37,38^, and we have also reported previously fabrication of highly functionalizable non-woven mat using our azido-TPU^26^. Obviously, SP-TPU can be also adopted in electrospinning without any tedious optimization process as indicated in Fig. 7a. Because SP-TPUs with different concentration of organic dyes can be introduced, non-woven patches with different chromic after UV exposure are obtained as illustrated in Fig. 7b. These chromic patches with different signal via same external stimuli - in other words, one can also generate certain level of signals via different external stimuli by controlling concentration of dyes or adopting different dyes - can be expanded to detect quantitative level of signal.

Previous works for these type of applications are only depended on (a) surface treatment of chromic dyes onto polymeric structures or (b) composite formation with polymers. However, these methods have several limitations; (a) only limited amount of dyes can be introduced via surface treatment and b) there should be elusion issue from matrix, resulting in less long-term or environmental stability. SP-TPUs (or TPU with different chromic dyes) can overcome above drawbacks by covalently substitution of dyes on polymer. In addition, because the concentration of dyes (again the level of chromic) are pre-defined, better reliability can be guaranteed in practical applications. As demonstrated in Fig. 7c,d, the color change of SP-TPU is totally reversible and spatial selective against stimulus (here UV). It is totally supporting that these chromic sensors can be reusable and useful in some applications, such as fingerprint detection via humid-chromic^39,40^.

**Figure 8:**
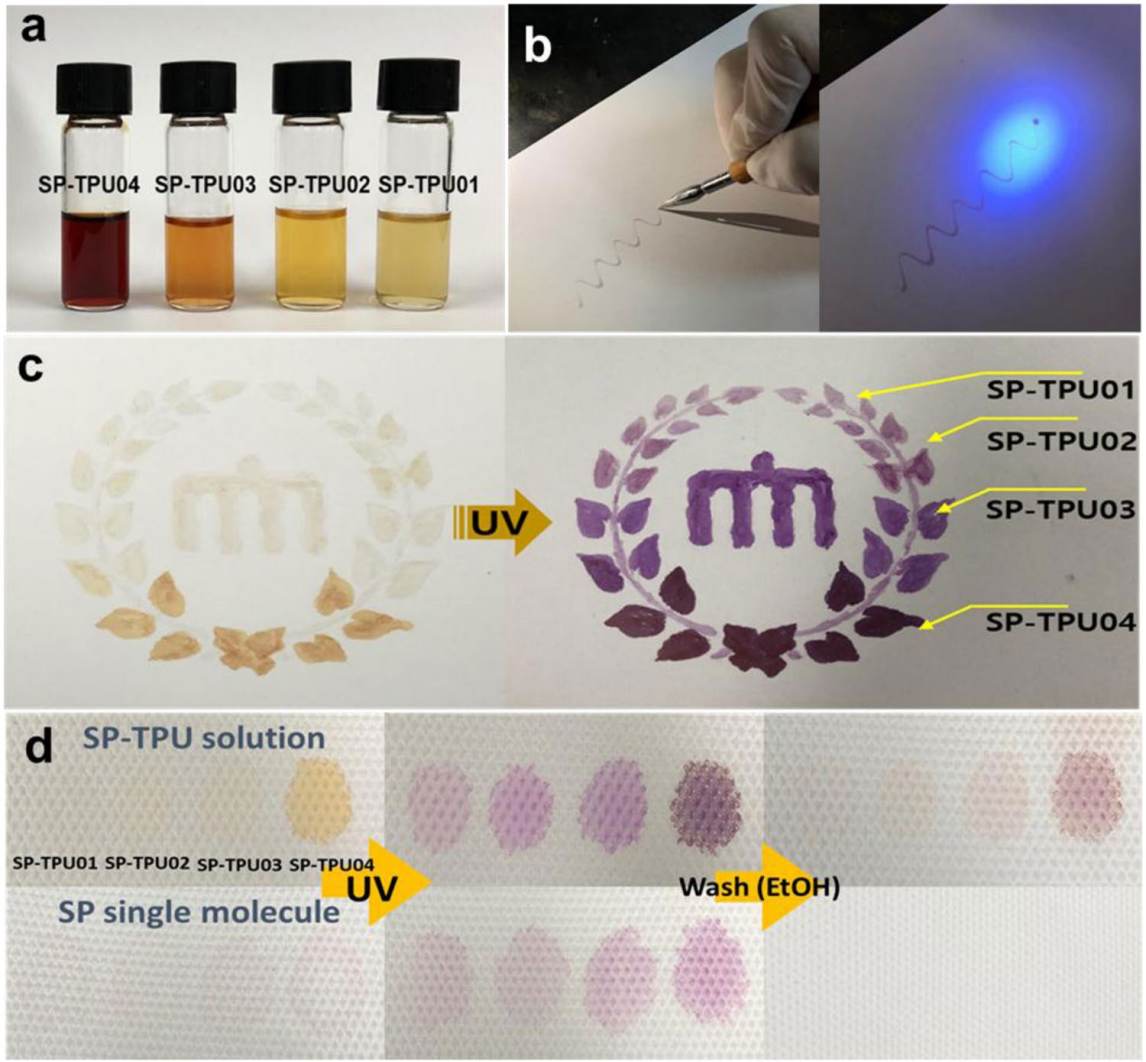
Inking process: (**a**) polymeric solution with four different type of SP-TPU, (**b**) ink writing of SP-TPU with fountain pen, (**c**) coloring with four types of concentrations inks before and after UV irradiation and (**d**) SP-TPU secret coding on paper with environmetal and washing resistance.

For easier utilization in practical fields, these SP-TPU can be commercialized in the form of organic solution as well. Based on their great solubility, SP-TPU with different amount of dye can be formulated in the form of sol-ink (Fig. 8a). Because the dye concentration on polymers are pre-defined again, these inks can be directly applicable without further treatment to prepare chromic drawing or pattern via writing or ink-jetting. As a representative example, the SP-TPU inks are introduced into fountain pen as illustrated in Fig. 8b. These types of pen can be utilized to draw letters or color pictures which is only shown-up after UV irradiation (Fig. 8b,c). Different level of colors is also possible to generate as shown in Fig. 8c, so that one can do coloring a pictures with different type of chromic. In addition, these chromic inks also are applicable in anti-forgery for banknote. As denoted in Fig. 8d, certain combination of circle codes (here, dot-secret codes with 4 different levels of color in order) can be imprinted onto money-paper via ink-writing, which is shown-up under UV-irradiations reversibly. The color of these chromic-polymers are predefined at a synthetic level, and the synthetic procedure is difficult to follow, so that it can be helpful to prevent counterfeit money. In addition, these inks are basically polymers differently to single organic molecules of chromic dyes, and thus it is much more beneficial for long-term stability and washing resistance as shown in Fig. 8d.

## Conclusion

In conclusion, polyurethane anchored with chromic dyes are synthesized and provided as generic platform for chromic applications. Chromic dyes such as SP can be covalently bonded on highly functionalizable TPU with abundant azide groups via controlled and tailored concentration with maintaining their processibility. Based on their excellent solution properties, these SP-TPUs can be formulated into diverse type of polymeric products through conventional solution processes; films, fibers, non-woven mat and sol-ink, etc. The chromic properties are maintained and the level of color can be manipulated in pre-defined, intended manner. These polymeric products have great potential to be applicable in diverse fields of practical applications. Most of important feature of these methods compared to previous single molecular chromic dyes will be the integration of chromic dyes and carriers simultaneously, so that it is possible to use these directly at the fields. These series of methods can be also further expanded by combining with different type of chromic dyes in near future.

## supplementary material

Supplementary Figures
