# Supplementary material for: Dye Clicked Thermoplastic Polyurethane as a Generic Platform toward Chromic-Polymer Applications
Source: Sci Rep. 2019 Dec 9;9:18648. doi: 10.1038/s41598-019-54832-5 (PMC6901444; doi:10.1038/s41598-019-54832-5)
Supplement: Supplementary file 1 — Supplementary Figures [file 41598_2019_54832_MOESM1_ESM.docx]

*Supporting information*

Dye Clicked Thermoplastic Polyurethane as a Generic Platform toward Chromic-Polymer Applications

Eunbyeol Seo ^a^, Jihyun Choi ^a^, Bumjae Lee ^a^, Young-A Son ^b^ and Kyung Jin Lee ^a *^

^a^Department of Chemical Engineering and Applied Chemistry, College of Engineering, Chungnam National University, 99 Daehak-ro (st), Yuseong-gu, Daejeon 305-764, South Korea

^b^Department of Advanced Materials Engineering, College of Engineering, Chungnam National University, 99 Daehak-ro (st), Yuseong-gu, Daejeon 305-764, South Korea

Keywords: thermoplastic polyurethanes(TPU), chromic dye, spiropyran, click chemistry, polymeric solution processes


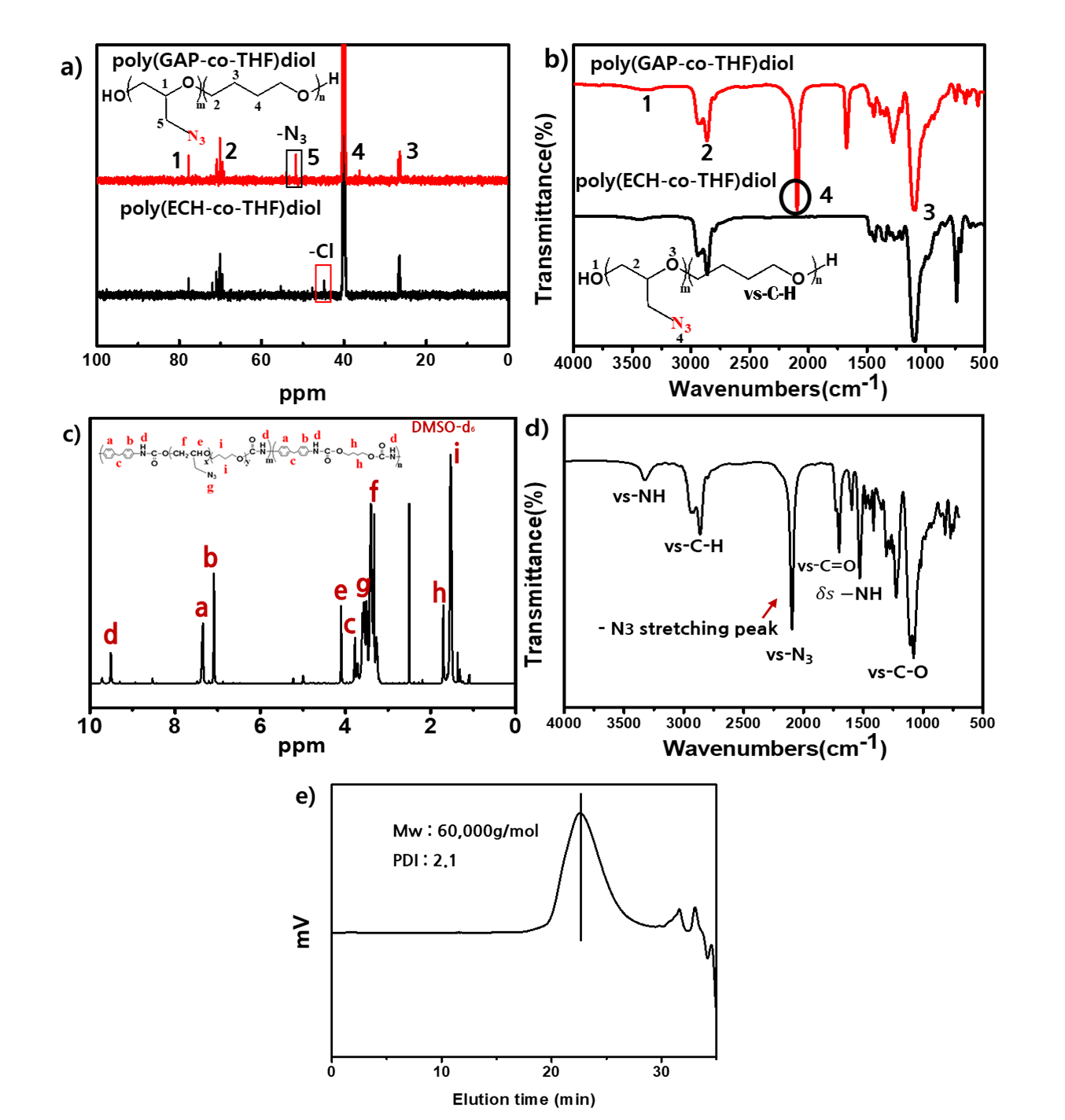


**Figure S1.**  Confirmation of poly (GAP-*co*-THF)diol from poly (ECH-*co*-THF)diol: a) ^13^C-NMR analyses FTIR and b)  FTIR, analysis of azido-TPU polymer: c) ^1^H-NMR spectrum, d) FTIR spectrum and e) GPC curve of azido-TPU.

**Figure S2.**  GC-Mass spectrum of alkyn functionalized SP dye.

**Figure S3.**  PL spectra of SP dye (single molecule) solution with different concentration (normalized intensity).


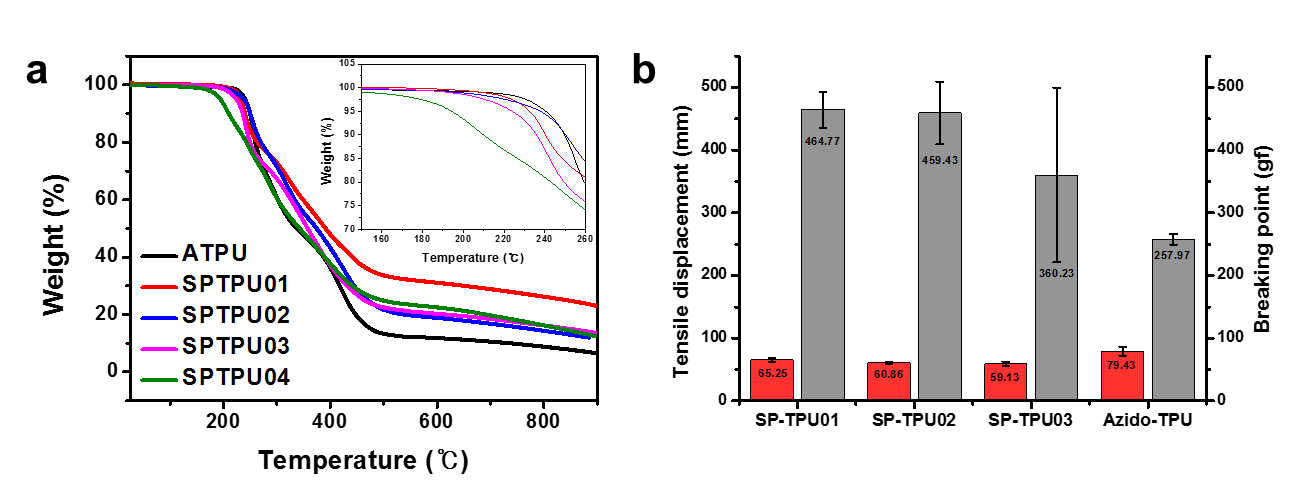


**Figure S4.** a) Thermal analysis of azido-TPU and each of SP-TPU polymer, b) mechanical properties of each polymers (red: tensile displacement, grey: breaking point)


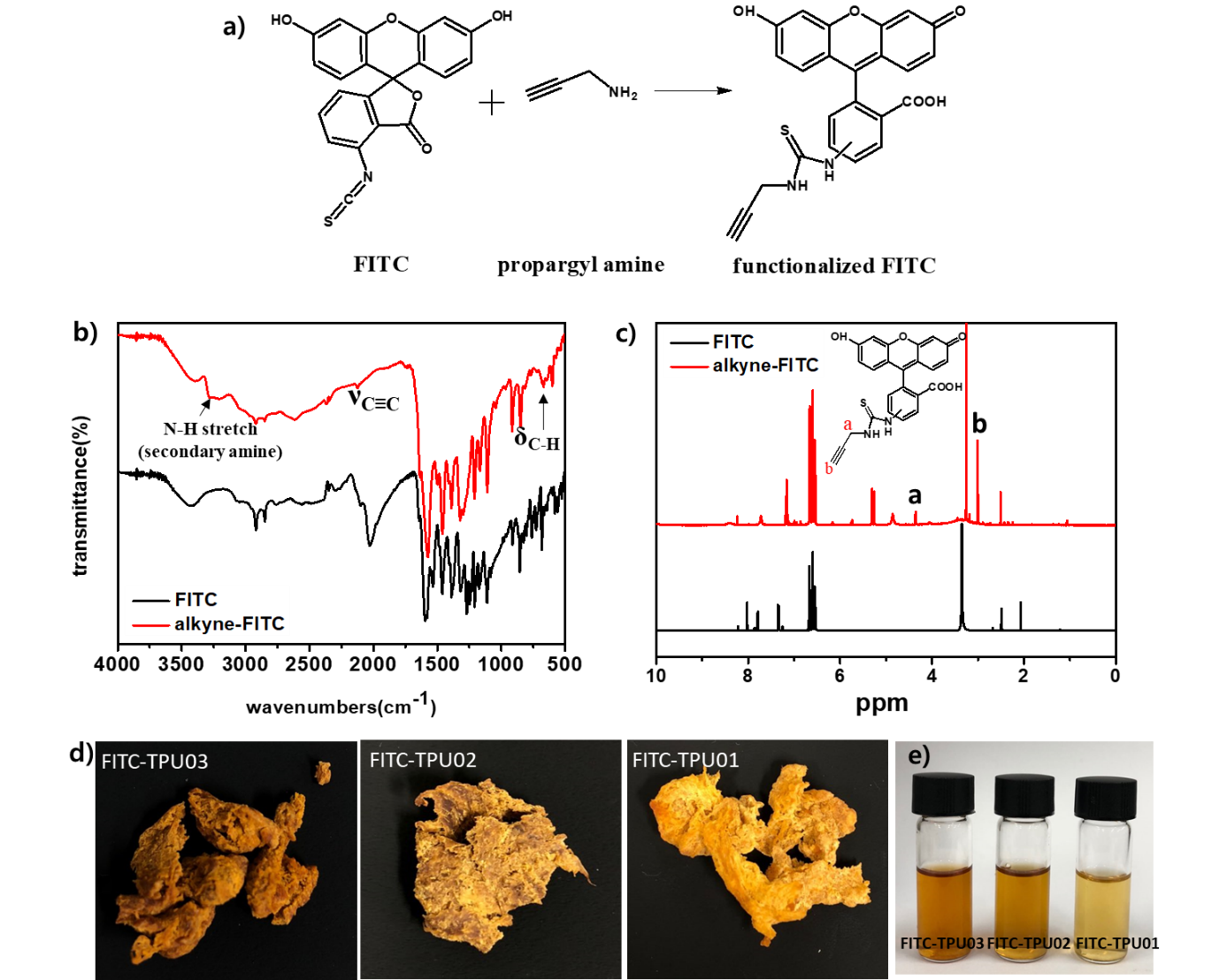


**Figure S5.**  a) scheme of functionalized FITC, b) FTIR spectra, c) ^1^H-NMR spectra and d) photograph of three concentrations FITC-TPU polymers and e) polymer solution images ith three concentrations


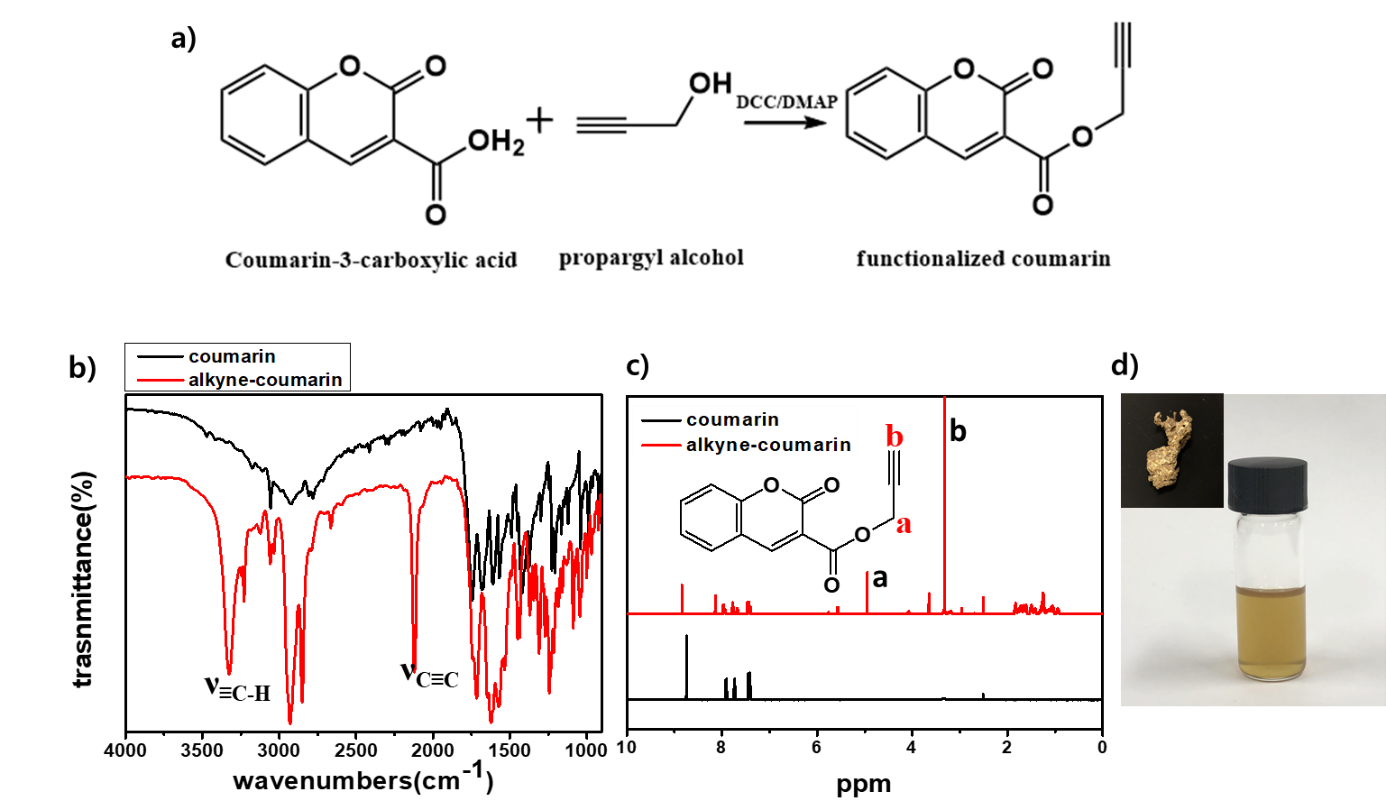


**Figure S6**  a) scheme of functionalized coumarin, b) FTIR spectra, c) ^1^H-NMR spectra and d) photograph of coumarin-TPU polymer solution
